# Lung Cancer and Human Papilloma Viruses (HPVs): Examining the Molecular Evidence

**DOI:** 10.1155/2012/750270

**Published:** 2012-01-26

**Authors:** Priya R. Prabhu, D. Jayalekshmi, M. Radhakrishna Pillai

**Affiliations:** Cancer Research Program, Rajiv Gandhi Centre for Biotechnology, Thiruvananthapuram 695014, Kerala, India

## Abstract

Human papilloma virus (HPV), known to be an etiological agent for genital cancers, has been suggested also to be a possible contributory agent for lung cancer. Alternatively, lung cancer, formerly considered to be solely a smoker's disease, may now be more appropriately categorised into never smoker's and smoker's lung cancer. Through this paper we attempt to bring forth the current knowledge regarding mechanisms of HPV gaining access into the lung tissue, various strategies involved in HPV-associated tumorigenesis in lung tissue.

## 1. Introduction

The World Health Organization (WHO) states lung cancer to be the most common cause of cancer-related deaths worldwide (1.3 million/year). The most important and common cause of lung cancer is the long-term exposure to tobacco smoke. Nevertheless, the global burden of 15% of men and 53% of women with lung cancer, which is not attributable to tobacco smoking, amounts up to 25% of all lung cancer cases worldwide [1]. That would position lung cancer in never smokers among the top ten of the causes of cancer-related deaths worldwide. Suggested risk factors for the disease in never smokers include environmental tobacco smoke, radon gas, cooking oil vapours, indoor coal and wood burning, asbestos, genetic factors, and viral agents.

Syrjänen in 1979 first suggested the possibility of human papilloma virus (HPV) involvement in bronchial squamous cell carcinoma [2]. Several studies after that, put together, further suggest strongly a role for HPV as an aetiological agent of lung cancer. Starting from the preliminary step of HPV gaining access to the lung tissue, discrete molecular and genetic changes have to occur for tumor initiation and progression. However, studies on HPV and lung cancer are very limited, of which epidemiological reports outnumber molecular mechanisms. There are several questions to be answered to get a complete picture of any possible mechanism for HPV-induced lung cancer. This paper tries to summarize work carried out in this field and dwells into possible mechanisms of molecular pathogenesis of HPV-induced lung cancer. 

## 2. HPV and Carcinogenesis

Human papilloma viruses, belonging to the Papillomaviridae family, are DNA viruses and are strictly host specific and exquisitely tissue tropic, having a preference to infect cutaneous or internal mucosal surfaces. Nearly 200 subtypes of HPV have been identified which are categorized as high risk (HR) and low risk (LR) based on their oncogenic potential. HPV infects the keratinocytes found in the basal layer of the skin (stratum germinativum). Several investigations have convincingly proved the presence of human papilloma virus in the lesions of upper aerodigestive tract (UADT). We have previously described the distribution of HPV types in oral cancer [3] as well as in the larynx [4]. The process of HPV-induced cell transformation is a combined manifestation of several discrete cellular, genetic, and molecular alterations accumulated in the mucosal tissue, termed “condemned mucosa syndrome,” which later progresses onto invasive cancer [5]. The viral proteins E6 and E7 contribute predominantly to the process of carcinogenesis and further tumor progression. These oncoproteins interact with critical cell cycle regulators to hamper their activity ensuing deregulation of the cell cycle machinery leading to uncontrolled cell proliferation. The virally encoded E6 binds to a cellular ubiquitin/protein ligase, E6-AP, and to p53 resulting in ubiquitination of p53 leading to its proteolytic degradation [6]. On the other hand, the E7 oncoprotein binds to the pRb dissociating the transcription factor E2F from the pRb/E2F complex, resulting in the transcriptional activation of several genes which facilitate cell proliferation. Rb/E7 complex formation is important for E7-induced cell transformation [7].

## 3. HPV and the Lung

### 3.1. Why to Study HPV in relation to Lung Cancer? The Epidemiology

Roglić et al. in 1975 and Rubel and Reynolds in 1979 provided first evidence for involvement of HPV in benign bronchial lesions, through the observations of koilocytosis in sputum samples characteristic of HPV infections [8, 9]. Syrjänen described that the epithelial changes seen in bronchial carcinoma closely resembled HPV-induced genital lesions [2]. 

Klein et al., in 2008, have beautifully concised worldwide epidemiological data available in the HPV incidence in lung cancer [10]. The study suggests an incidence of 24.5% of HPV-associated lung cancers. The distribution pattern ranges from a prevalence of 15% in the American continent and 17% in Europe to a mean incidence of 35.7% in Asia. In majority of the studies, the high-risk subtypes of HPV detected in the lung cancer tissues were 16, 18, 31, and 33, and the most prevalent low-risk subtypes were HPV 6 and 11. The alarmingly high percentages of incidences reported in Greece (69%), Taiwan (78.5%), and Japan (79%) suggest HPV to be the second most important possible etiological agent of lung cancer after cigarette smoking and advocate the need for further research in this issue.

### 3.2. The Histological Fondness

The human respiratory tract has a divergent histology of the mucous lining with columnar epithelium throughout the respiratory tract and stratified columnar epithelium covering the mucosa of the pharynx and larynx. The resulting squamocolumnar junctions (SCJs) can be compared to a similar junction in the cervix and could favour establishment of HPV infections. Lung cancer can be histologically categorised, based on the biology, therapy, and prognosis as small-cell lung carcinoma (SCLC) and non-small-cell lung carcinoma (NSCLC). NSCLCs are further classified into squamous cell carcinoma (SCC), adenocarcinoma (ADC), and large-cell lung carcinoma (LCLC). Lung cancer in never smokers when considered as a different disease is more frequent in the NSCLCs than in SCLCs [11].

### 3.3. Mode of Transmission

HPV cannot bind to the live tissue, instead infects the epithelial tissue through the exposed basal keratinocytes following microabrasions of skin as would occur after a sexual intercourse. However, how a well-protected organ like lung deeply positioned in the thoracic cavity, where probability of acquiring abrasions and thereby getting exposed to HPV, is not clear. Multiple sex partners, oral-genital sex, and oral-anal sex could be some of the factors favouring transmission of HPV to the oral cavity and oropharyngeal cancers. Prevalence of oncogenic mucosal HPV is higher in younger age oral cavity or oropharynx cancer cases whose sexual practices are typically associated with sexual transmission of the virus [12].

The study results from two different cancer registries in the United States, Connecticut, and the Surveillance, Epidemiology, and End Results (SEER) program tumor registries report evidence of shared etiological factors of various second primary cancers following anal and cervical cancer. There were 275 cases of lung or bronchial cancer following cervical cancer recorded in these registries as compared with 91.8 expected cases, a relative risk of 3.0 (95% confidence interval 2.7–3.4) [13]. Studies conducted by Hennig et al. found that 49% of bronchopulmonary carcinomas were detected to be HPV DNA positive in the women who had a clinical history of CIN III lesions [14].

**Figure 1:**
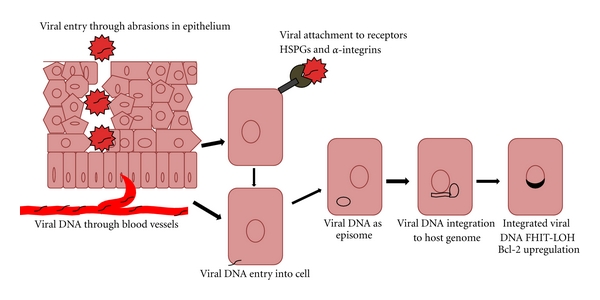
Viral entry and integration. The diagram shows the entry of viral DNA into the lung tissue either through the microabrasions in the membrane or through the haematogenous route from a different entry point as cervix or anus. Molecules such as HSPGs and *α*-integrins serve as surface receptors for the viral attachment and entry. Viral DNA exists and episome and later on integrates into the host genome losing an FHIT gene allele resulting in heterozygosity and subsequently upregulates the expression of proapoptotic protein Bcl-2.

HPV 16 and 18 E6 mRNA detected in the peripheral blood of cervical cancer patients [15] suggests a possibility that HPV infection in the lung tissue may originate in the cervix and later infect lung via a hematogenous spread (Figure 1)  [16]. It has also been suggested that the presence of circulating HPV 16/18 DNA may act as a risk marker for lung cancer [17]. Peripheral blood mononuclear cells (PBMCs) can function as HPV carriers and might spread the virus through blood. In all the samples tested positive, the HPV genome was found to be in the episomal form, although in a low copy number [18].

All these reports put together designate oncogenic HPV DNA as a possible risk factor in developing second primary cancers after HPV-related primary neoplasias. Whether or not persistent HPV infection or HPV-induced cervical cancer is a prerequisite for the development of HPV-associated lung cancer needs further investigation.

### 3.4. Entry of Virus into the Cells

The entry of HPV into the cells *in vitro* is initiated by binding to a cell surface receptor in contrast to the *in vivo* situation where the basement membrane is a primary site of virus binding [19]. Widely distributed and evolutionary conserved cell surface receptors, heparin sulphate, and stabilizing proteoglycans (HSPGs) are presumed to be epithelial cell receptors for HPV [20]. The existence of HSPGs on the cell surface, [21] as well as extracellular matrix [22] of lung fibroblasts, can make them suitable receptors for viral entry into lung tissue (Figure 1). As ubiquitous members of extracellular membrane (ECM) microenvironment and hence the cancer stem cell niche, HSPGs are major factors responsible for the microenvironment changes, involved in the tumor initiation, progression, and malignant conversion [23]. HSPG functions as an attachment factor in HPV infection, and the resulting interaction promotes essential conformational changes in viral capsid, but HSPGs are obviously not the cell surface receptors that arbitrate virion internalization and the subsequent events in infection. The cell adhesion receptors *α*6 integrins have close association with HSPGs as matrix components, and act as secondary receptors for HPV after its interaction with HSPG [19]. The drug surviving side population cells from human lung tumor tissue express the *α*6 integrin otherwise termed as very late antigen-6 (VLA-6) [24]. The presence of CD49f receptors (another term for *α*6 integrins) in the adult mouse lung stem cells have also been demonstrated [25] all suggestive of the possible involvement of lung cancer stem cells in the HPV entry leading to infection and cancer. The integrins, heterodimeric glycoproteins comprising of *α* and *β* subunits, are expressed in a variety of cell types, primarily involved in cell-matrix and cell-cell interactions, act as virus receptors, and aid in initial binding and/or internalization of viruses into the host tissue. Of the various *α* and *β* subunits known, *α*6, *β*4, and *β*1 play a major role in the HPV binding and internalization. The preferential expression of *α*6 subunit with the *β*4 subunit forming the *α*6*β*4 complex expressed exclusively in the stratified squamous epithelium [26], the primary sites of HPV infection, makes *α*6*β*4-integrin the key receptor for HPV binding and possibly accounts for the predominance of HPV in squamous cell carcinoma than in other histologic subtypes of lung cancer.

### 3.5. Presence of HPV DNA in the Lung Cancer Tissue: Proof for a Causal Association

Although the presence of oncogenic HPV in the lung tumor tissue has been reported in several studies, the causal association of the HPV and lung cancer needs to be authenticated with evidence. An argument supporting the causative role of HPV in the tumorigenesis of lung tissue is that HPV DNA is indeed integrated into the host genome, which is the initial key step in the tumor initiation [27]. Furthermore, the viral oncoproteins E6 and E7 are in fact found to be expressed in the lung tumors, down regulating tumor suppressor genes such as p53 [28]. Taiwanese nonsmokers had significantly high prevalence of HPV16/18, suggesting HPV infection as a possible etiological agent of lung cancer in nonsmokers. Moreover, the nonsmoking females are more prone to HPV positivity than their male counterparts. Also, compared to the male smokers, the female nonsmokers are at a higher risk of having HPV in their lung tumor tissue [16]. The reason for this gender-based prevalence is yet unidentified. Though a few studies reported HPV absence in nonsmokers with lung cancer, this can be attributed to the technical variations in the experimental conditions such as specificity of the primers and probes used, disparity in the study subjects, and above all the overall prevalence of HPV in the study population.

## 4. HPV-Induced Lung Tissue Carcinogenesis: Possible Mechanisms

The process of HPV-mediated carcinogenesis is the consequence of an integrated process of defective apoptosis, neovascularisation, and cellular immortality [3]. Once the pathogen gains entry into its tissue of interest, it takes up the cellular machinery, replicates its genome, evades cell apoptosis, and initiates tumor formation. A few possible molecular events manifested upon HPV infection in addition to the already reported ones in the HPV-induced lung tumor tissue are summarised here.

### 4.1. FHIT LOH

Once inside the cells, HPV genome integrates into the host genome. The fragile site FRA3B, adjacent to the fragile histidine triad (FHIT) is a site of integration of HPV genome. The FHIT gene located in chromosome 3p14.2 undergoes frequent allele loss of heterogeneity (LOH) or homozygous deletions in several cancers including lung cancer. Upon integration of HPV to this site, allele loss can occur to the FHIT gene (Figure 1). A study conducted among HPV infected, nonsmoking, female lung cancer patients from Taiwan suggested that tumorigenesis may be in part due to the increase in frequency of the FHIT LOH, reducing the expression of FHIT [29].

### 4.2. Bcl-2 Up-Regulation

The status of HPV in the tissue may determine expression level of the proapoptotic protein Bcl-2, the levels of which being significantly higher in integrated HPV positive patients than those that were negative or with episomal forms (Figure 1) [30].

### 4.3. Ras Gene Mutations

**Figure 2:**
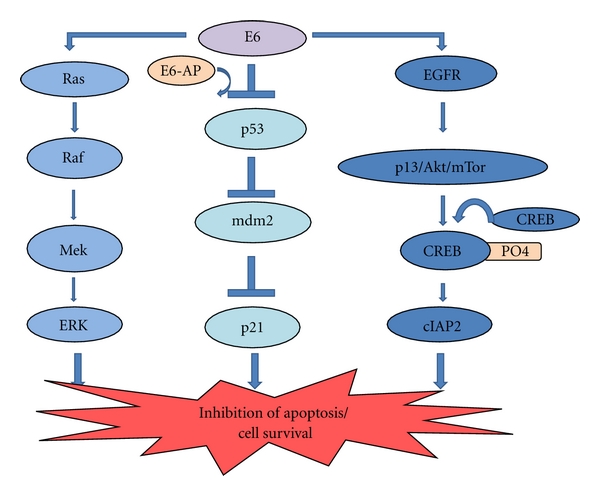
E6-mediated survival signaling. The figure summarizes the different survival signalling pathways activated by the viral protein E6. While it activates the mitogenic signals in the Ras/Raf/Mek pathway, E6 blocks the p53 activation in turn inhibiting the action of p21, all ultimately inducing cell survival. E6 also upregulates the expression of epidermal growth factor receptor (EGFR) and inhibits apoptosis by the activation of an inhibitor-of-apoptosis protein (cIAP2) thereby obstructing apoptosis.

One of the most important molecular changes documented in lung cancer progression is activation of dominant oncogenes such as ras family genes. Of various genes in the ras family, k-ras gene is found to have point mutations in codon 12, in most of the lung carcinomas. Also, in 50% of HPV positive lung tumors, k-ras mutation was seen to coexist, constitutively activating the Ras/Raf/Mek pathway (Figure 2). This suggests that HPV infection is not sufficient by itself for malignant transformation but requires cooperation of the activated ras gene [31].

### 4.4. Does the E6 and E7 Genes Have the Same Role and Importance in Lung Cancer Pathogenesis as They Have in Cervical Cancer?

#### 4.4.1. E6-p53

The E6 protein has a well-recognised signature in pathogenesis of HPV-induced cervical and oral cancer. E6 is the major viral protein which can activate two independent pathways to prevent apoptosis-p53-dependent and p53-independent pathway [32]. Relationship between E6 and p53 has been conclusively shown in HPV-infected cells [33]. E6 protein binds to the E6-AP, a host cell ubiquitin ligase, and p53 tumor suppressor protein simultaneously and induces accelerated proteosomal degradation of p53 (Figure 2) [6].

Kinoshita et al. in 1995 reported expression of E6 mRNA in the HPV 18 DNA-positive lung tumor tissues [34]. In this study, they also put forward the possibility of cellular targets of HPV other than p53. HPV E6 proteins are indeed expressed in lung tumor tissues, which are negatively associated with the p53 expression. The corresponding downregulation of mdm2 and p21 (Waf1/Sdi1/Cip1), which are the downstream targets of p53, leads to the conclusion that HPV E6 protein can inactivate the expression of p53 in lung tumor tissues (Figure 2) [27]. The human Dead-box RNA helicase (DDX3), which plays a role in regulation of gene expression via RNA metabolism, transcription, splicing, mRNA export, and translation, has been associated with development of viral associated cancers. DDX3 transcription is predominantly regulated by p53, and p21 transcription is being synergistically suppressed by the alteration of the p53-DDX3 pathway via E6, in lung tumors. Reduction of p21 make lung cancer patients vulnerable to tumor reversion and thereby bring about poor relapse-free survival [35].

#### 4.4.2. E6-cIAP

The viral oncoprotein E6 can also promote carcinogenesis by upregulating expression of inhibitors of antiapoptosis proteins (IAPs). Inhibitors of antiapoptosis proteins (IAPs) directly inhibit caspases to block cell apoptosis. HPV E6 upregulates the expression of cIAP2 via epidermal growth factor receptor (EGFR)/phosphatidyl inositol 3-kinase (PI3K)/AKT cascade. CREB, which is a regulatory targeting molecule of AKT, and is phosphorylated via the EGFR/PI3K/AKT pathway, which plays a crucial role in the upregulation of cIAP2 by E6 protein (Figure 2). This upregulation has been shown to confer resistance to cisplatin in HPV 16/18 infected lung cancer [36].

#### 4.4.3. E6-Bak

The Bcl-2 homologous antagonist/killer (Bak) protein is a proapoptotic member of the Bcl-2 gene family which is involved in apoptosis. In the p53-independent pathway, E6 protein binds the Bak protein and degrades it. The proapoptotic effect of Bak is a main target of HPV E6 protein. E6 from high-risk HPV subtypes (16,18) and low-risk type (11) can bind to Bak, *in vitro*, and can stimulate its degradation, *in vivo*. Bak can associate with E6-associated protein in the absence of E6 in contrast to p53, and the subsequent inhibits the Bak-induced apoptosis through this E6-AP-dependent process [37]. Though the status of Bak expression in HPV-induced lung cancer is yet to be studied, we strongly suspect this mechanism to be active in promoting the tumorigenesis of lung tissue.

#### 4.4.4. E7-pRb

**Figure 3:**
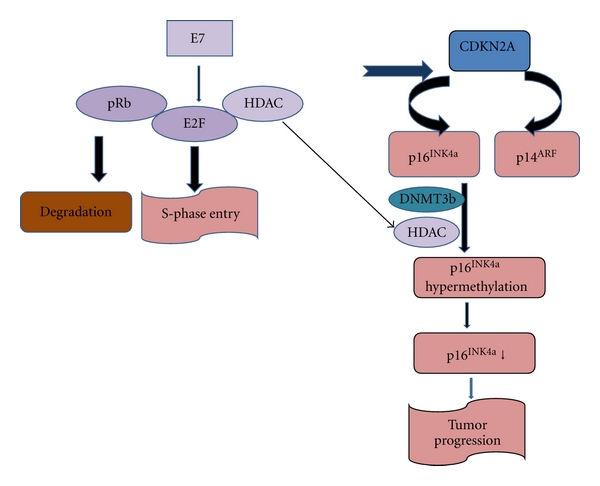
E7-dependent tumorigenic signaling. Diagram summing up the steps targeted by the viral protein E7 in the tumorigenesis of the lung tissue. The histone deacetylases (HDAC) gets released from the retinoblastoma protein (pRb), elongation factor (E2F), and HDAC complex when E7 binds to it and leads to the downregulation of tumor-suppressor-p16^INK4^ A protein by hypermethylation thus leading to tumorigenesis.

The coexpression of E7 along with the E6 is indispensable for the increased oncogenic activity of the HPV. The E7 protein of the high-risk HPV has a strong affinity to bind to pRb, abrogating the pRb signalling pathway. Kinoshita et al. in 1995 reported the coexpression of E7 mRNA along with E6 mRNA in the HPV 18 DNA-positive lung tumor tissue [34]. The cell cycle regulatory gene CDKN2A regulated by pRb is found to be altered in the HPV associated tumors. Of the two proteins encoded by CDKN2A, p16^INK4a^ and p14^ARF^, the former prevents the cells from S-phase entry by inhibiting CDK4/6-mediated phosphorylation of Rb and the p14^ARF^ is a p53 stabilizer. p16^INK4a^ gene is epigenetically modified by CpG island promoter hypermethylation, thus silencing it. HPV E7 inactivates the pRb, releasing the histone deacetylases (HDAC) from the E2F-Rb-HDAC complex to enhance p16^INK4a^ hypermethylation through chromatin remodelling by HDAC (Figure 3). Enzymes named DNA methyl transferases (DNMTs) mainly of three types (DNMT1, DNMT3a, and DNMT3b) by their complex interplay establish the cytosine methylation pattern. The p16^INK4a^ promoter hypermethylation was analysed using methylation-specific PCR (MSP), the results of which showed p16^INK4a^ hypermethylation in 59.7% of smoking males, 36.6% of nonsmoking males, and 60.3% of nonsmoking females lung tumors among Taiwanese population [38]. p16^INK4a^ hypermethylation frequency in nonsmoking female lung tumors with HPV infection was as high as 70% compared to those without HPV infection, which was as low as 30% [38]. A statistically significant correlation was observed only in the nonsmoking female lung cancer cases (*P* = 0.017), but not in male smoking or nonsmoking lung tumors. Furthermore, reports from the same research group demonstrated involvement of HPV infection in increased expression of the DNMT3b (DNA methyl transferase), to cause p16^INK4a^ promoter hypermethylation among the nonsmoking female lung tumor (Figure 3) [39].

### 4.5. Role of E5 Protein of HPV in the Transforming Activity

**Figure 4:**
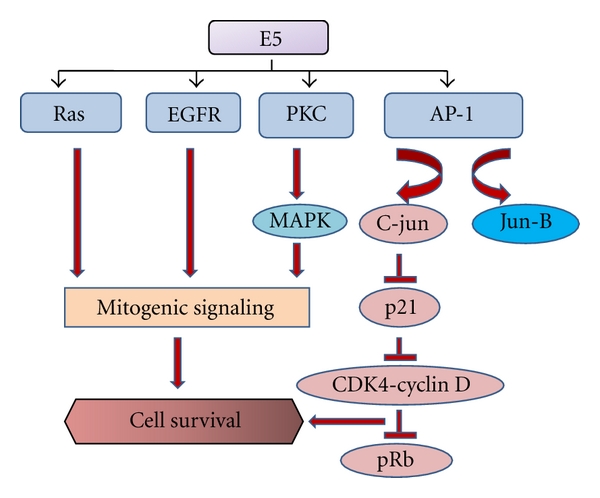
Possible role of E5 protein in tumor initiation. This figure condenses the multiple pathways that are suspected to be activated by viral protein E5 leading to cell survival. The mitogenic signaling downstream of Ras, epidermal growth factor receptor (EGFR) and protein kinase C (PKC) are turned on by the E5 protein. Additionally, E5 can also activate the activator protein-1 (AP-1), which through c-jun may result in cell survival.

The HPV E5 viral protein has been shown to have weak transforming activity. Once the HPV genome is integrated into the host genome, the E5 gene is frequently deleted, and hence the E5 gene expression is terminated during the progression of disease from the low grade to malignant stage [40, 41]. So if at all E5 protein has an effect on the process of carcinogenesis, it has to act as an early-stage protein, that is, before the HPV genome integration. The key activity of HPV E5 seems to be the upregulation of EGF receptors in a ligand-dependent manner. The E5 protein controls mitogenic signalling pathways by forming complex with EGFR [42, 43]. This complex formation enhances the activation by EGF [43]. HPV E5 exerts its action by upregulation of the EGF receptor in a ligand-dependent manner. The mechanism by which the HPV E5 does this appears to be due to (i) a decrease in the rate of receptor degradation mediated by binding of E5 to the 16 kDa subunit of the endosomal proton pump ATPase and (ii) a net increase in the receptor phosphorylation without decreased downregulation [44, 45]. E5 protein can also activate MAP kinases via PKC (protein kinase C) and Ras-mediated receptor tyrosine kinase (RTK) pathway. The E5 protein can activate the nuclear oncogenes, c-jun and jun-B, through the activation protein-1 (AP-1) binding site [46]. The activity of tumor-suppressor gene p21 (Waf1/Sdi1/Cip1) is being repressed through the activation of c-jun by the HPV E5 protein, which promotes cell proliferation [47]. Suppression of p21 gene by HPV E5 activates the CDK4-cyclin D complexes, which in turn phosphorylates pRb and inactivates pRb checkpoint control leading to uncontrolled cell replication (Figure 4).

The fact that the EGFRs are often upregulated in the lung tumor tissues highlights the possibility of the E5 protein playing a role in the early stages of the transformation process. On the contrary, Crusius et al. reported that the HPV 16 E5 protein modulates the stress-dependent activation of ERK1/2 and p38 MAP kinase activation in human keratinocytes by an EGF-independent mechanism [48]. No studies are reported yet, indicating the presence of HPV E5 protein in the lung tumor tissues. Studies towards this aspect would probably suggest new vaccine candidates or therapeutic targets against HPV-induced lung cancer.

### 4.6. Does ER-EGFR Crosstalk Favour HPV Persistence Leading to Tumorigenesis? HPV-Induced Lung Cancer Is More Prevalent in Females Than in Males

**Figure 5:**
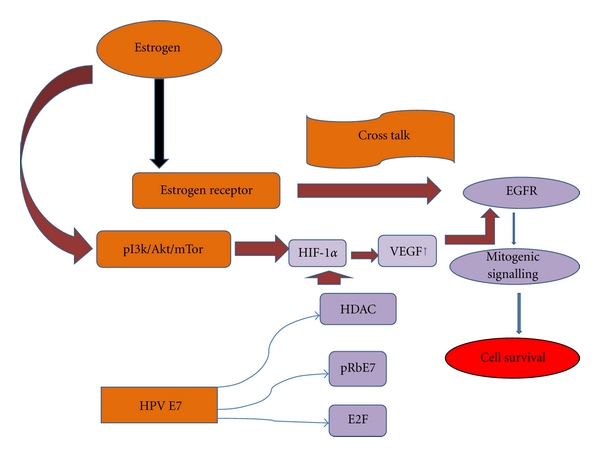
Possible estrogen-EGFR-HIF-1*α* crosstalk. Diagrammatic representation of the possible crosstalks between estrogen, epidermal growth factor receptor (EGFR), and hypoxia-inducible factor-1*α* (HIF-1*α*) activating the mitogenic signalling resulting in the cell survival.

Estrogen contributes to a large extent to the onset of HPV infection and tumor progression [49]. Though the presence of estrogen receptors (ERs) in human lung tissue has been controversial for many years, it has been proved beyond doubt that some forms of both ER*α* and ER*β* are indeed present in normal lung cells as well as in lung tumors, which are undeniably functional, as evidenced by the estrogen-induced cell proliferation in the lung *in vitro *as well as *in vivo*; activation of transcription from estrogen response elements (ERE) in lung cancer cells by estrogen and estrogen-stimulated secretion of a growth factor thought to be involved in lung tumorigenesis, collectively demonstrating that ERs play a biological role in the lungs [50]. Aromatase, the enzyme involved in catalysing the biosynthesis of estrogen, is induced in cervical carcinomas favouring the increased expression of viral oncogenes E6 and E7 [51]. This is expressed in lung tissue also which catalyses the local production of estrogen from androgen and is suggested to be a predictive biomarker for the prognosis of NSCLCs [52]. This enhanced local site production of estrogen triggers the nongenomic mechanism of estrogen action, which rapidly activates EGFR. The well-established crosstalk between ER and EGFR in head and neck cancers [53] arbitrates this action which is further validated by the colocalised membrane ER and EGFR in the lung tumors. Combined targeting of ER and EGFR in NSCLC has been proved to enhance antiproliferative effects in the treatment of lung cancer [54]. We strongly argue that this ER-EGFR crosstalk could occur in the lung tissue which can consequently favour HPV persistence and malignant transformation of the lung tissue (Figure 5). Furthermore, when this probable crosstalk fits in position, the increased vulnerability of female never smokers to develop HPV-induced lung cancer as well as the histologic affinity of HPV to NSCLC is better explained.

### 4.7. Estrogen—Hypoxia-Induced Factor—EGFR Crosstalk

Hypoxia-inducible factors (HIFs) are heterodimeric transcription factors that respond to changes in available oxygen in the cellular environment, specifically, to decreases in oxygen, or hypoxia by promoting the transcription of several hypoxia inducible genes. HIFs are frequently upregulated in solid tumors and are found to be expressed in three isoforms—HIF-1, HIF-2, and HIF-3— each with an *α* and a *β* subunit. Estrogen can regulate the HIF-1*α* expression through PI3K-Akt pathway (Figure 5) [55]. The oncogenic protein E7 of HPV props up HIF-1*α*-dependent transcription by blocking the interaction of HDACs with HIF-1*α* in a manner dependent on the HDAC binding domain of E7. The E6 protein primarily blocks the inhibitory effects of p53 on HIF-1 activity [56]. HIF-1 induces expression of the genes essential for adapting to hypoxia including those for erythropoietin, glucose transporters, glycolytic enzymes, and vascular endothelial growth factor (VEGF), eliciting successful homeostatic regulation under hypoxic conditions. In cervical cancer, VEGF upregulates EGFR and downregulates IGF-BP3, thus amplifying the cell proliferative activity of EGFR (Figure 5). This action of VEGF seems to be mediated, directly through EGFR or indirectly through HPV-E6 in the HPV-positive cancers [57]. EGFR upregulation triggers the downstream mitogenic signals supporting tumorigenesis. We strongly suspect this mechanism to be active in HPV-infected lung tissue which favours angiogenesis and metastasis.

## 5. Therapeutic Strategies

Would the vaccines such as Gardasil, Cervarix, and so forth, directed towards the cervical cancer ensure protection against HPV-induced lung cancer too? This would be a focal question of interest that needs to be addressed to combat the disease prophylactically. Given that the HPV subtypes infecting and most of the molecular events occurring in the cervical cancer may be extended to HPV-induced lung cancer, these vaccines can presumed to be effective against lung cancer too.

Lung cancer in nonsmokers is molecularly different disease from that seen in the smoker population. Mutation frequency as well as profile of the genes encoding EGFR, p53 and k-ras is conspicuously dissimilar in smoking and nonsmoking groups. Also, the HPV infection paves way to altering the signalling pathways leading to cancer. The fact that EGFR has a crucial role in lung carcinogenesis makes it an admirable target for therapy. Monoclonal antibodies targeted against EGF receptors, small molecule TK inhibitors, and antisense oligonucleotides can reduce the activity of EGFR thereby inactivating the mitogenic signalling pathways like Ras/Raf/Mek and PI3k/Akt/mTor pathway. One such anti-EGFR monoclonal antibody being used is cetuximab, in combination with chemotherapeutic agents such as Carboplatin and Paclitaxel, when provided concurrently or sequentially gave positive results in clinical trials [58–60]. Other than these, matuzumab which is a human monoclonal antibody, along with chemotherapy and certain other multikinase inhibitors, can be used in the treatment of advanced non-small-cell lung cancer [61]. Tyrosine kinase inhibitors such as Gefitinib interrupt the downstream signalling activated during the binding of ligands or mutational activation of EGFR [62]. Combined targeting of estrogen receptor and EGFR, using fulvestrant and gefitinib has proved to have enhanced antiproliferative effects in the *in vivo* studies [55]. Combination therapy targeting HIFs and EGFR would probably also enhance the treatment outcome.

MEK, a mitogenic signalling pathway protein activated as a result of k-ras mutations in HPV infection is another, suitable target for therapy. Ci-1040 difluorobenzamide is a MEK inhibitor which has been clinically tested in NSCLC patients [63]. Inhibitors of mammalian target of rapamycin (mTor), a protein kinase which regulates cell growth by regulating different cell processes: Rapamycin and Temsirolimus, gave partial response in patients with NSCLC [64].

## 6. Conclusion and Future Perspectives

From the reported studies and suspected crosstalks, we robustly argue EGFR to be a core molecular hub in pathogenesis of HPV-induced lung cancer. It can be presumed that combination therapy targeting EGFR and other molecules having crosstalk with EGFR may be effective in treatment of the disease rather than a single target therapy. It is still not apparent whether HPV is a causal factor of lung cancer or whether it is just an opportunistic pathogen in the lung cancer tissue and the exact molecular mechanisms behind it. Almost all of the signalling pathways having a role in lung cancer are found to be altered or blocked by human papilloma viral proteins initiating tumorigenesis. Further evidence is mandatory to substantiate beyond doubt the causative role of HPV in the lung tissue tumorigenesis. Moreover, the cofactors supplementing the HPV in the transformation processes are yet to be classified. Studies directed towards these targets would give a clearer image of the disease which will eventually pave the way to designing new prophylactic or therapeutic strategies in combating the disease.
